# Rewiring the proteome of the *Euscelidius variegatus* holobiont in response to Flavescence dorée phytoplasma

**DOI:** 10.1038/s41598-025-30920-7

**Published:** 2025-12-04

**Authors:** Simona Abbà, Marta Vallino, Simona Cirrincione, Cristina Lamberti, Beatrice Aiuto, Francesco Romaniello, Luciana Galetto, Cristina Marzachì, Domenico Bosco, Marika Rossi

**Affiliations:** 1 Istituto per la Protezione Sostenibile delle Piante, Consiglio Nazionale delle Ricerche, IPSP-CNR, 10135 Turin, Italy; 2 Istituto di Scienze delle Produzioni Alimentari, Consiglio Nazionale delle Ricerche, ISPA-CNR, 10095 Grugliasco, TO Italy; 3 Istituto Nazionale di Ricerca Metrologica, INRIM, 10135 Turin, Italy; 4 Dipartimento di Scienze Agrarie, Forestali ed Alimentari, Università degli Studi di Torino, 10095 Grugliasco, TO Italy

**Keywords:** Flavescence dorée, Genome assembly, Insect vector, DDA, DIA, Microbiome, Biological techniques, Biotechnology, Microbiology, Molecular biology, Plant sciences

## Abstract

The leafhopper *Euscelidius variegatus* is a laboratory vector of the phytoplasma associated to Flavescence dorée, a severe grapevine disease that threatens viticulture in Europe. Transcriptomic studies have already provided valuable insights into the mechanisms of insect-phytoplasma interactions, but proteomics can offer immediate insights into the cellular functions and metabolic adaptations of the insect and its microbiome to the presence of this plant bacterium. Here, the generation of new genomic data of the *E. variegatus* holobiont was instrumental in elaborating the first comprehensive proteomic profile of its response to Flavescence dorée phytoplasma (FDp). Both data-dependent acquisition and data-independent acquisition mass spectrometry were used to explore the complex molecular interactions between the insect host, its microbial community, and the phytoplasma. Results indicated a critical role of the insect mitochondria as a shared interface exploited by phytoplasmas for survival and propagation. Additionally, it appeared that the presence of FDp had a detrimental impact on the reciprocal metabolic support between the insect host and its two primary endosymbionts, predominantly resulting in a perturbation in amino acid synthesis and exchange. Proteins upregulated in response to FDp may represent promising targets for disrupting phytoplasma acquisition and transmission, either through rationally designed agrochemicals or gene silencing approaches.

**Supplementary Information:**

The online version contains supplementary material available at 10.1038/s41598-025-30920-7.

## Introduction

Insect pests pose a significant threat to global agriculture, due to direct damage as well as to their ability to transmit a broad range of plant pathogens. Plant bacteria and viruses can have a profoundly deleterious effect on crops, resulting in substantial economic losses through decreased yields, diminished product quality, and elevated costs for disease control and prevention. The Hemiptera order includes several of the most important insect families that act as vectors of plant pathogens, including phytoplasmas. These are wall-less, obligate bacterial plant pathogens that infect a wide range of economically important crops worldwide^1^. A notable example is Flavescence dorée (FD), a quarantine plant disease^2^ associated with the Flavescence dorée phytoplasma (FDp), which leads to severe vine decline and yield reduction across Europe. While the leafhopper *Scaphoideus titanus* Ball (Cicadellidae: Deltocephalinae) is recognized as the primary natural vector of FDp in vineyards^3^, the phylogenetically close leafhopper *Euscelidius variegatus* Kirschbaum is a key model vector to study the mechanisms of pathogen acquisition and transmission under laboratory conditions^4^. Such mechanisms, as well as feeding behaviors, immune responses and reproductive success, can be strongly modulated by the composition of the insect microbiome^5,6^. As is the case with a multitude of insect species, *E. variegatus* harbors a diverse microbial community, including both obligate and facultative symbionts. Its primary endosymbionts, “*Candidatus* Nasuia deltocephalinicola” (*Pseudomonadota,* hereafter *Nasuia*) and “*Ca*. Karesulcia muelleri” (*Bacteroidota,* hereafter *Sulcia*), play a critical role in its survival^7^. These obligate endosymbionts are located within specialized cells (bacteriocytes) in the insect body and supply the insect host with essential nutrients (e.g., amino acids, vitamins) that are typically absent from the insect phloem-sap diet^8–11^. In addition to these essential primary endosymbionts, *E. variegatus* may harbor a secondary, facultative, endosymbiont, initially known as ‘BEV’ and now classified as “*Ca.* Symbiopectobacterium sp” (*Pseudomonadota,* hereafter BEV)^12^. Unlike the obligate primary symbionts, BEV can be cultured in vitro*,* but its potential functional role in the biology and vector competence of *E. variegatus* is still under investigation. The genome of another symbiotic bacterium related to the *Asaia* species (*Pseudomonadota*) has been recently isolated from a laboratory rearing of *E. variegatus* and named *Sorlinia euscelidii*^13^. Furthermore, the iflavirus EVV1 has been identified as a stable component of the microbiome of the *E. variegatus* population reared in our laboratory^14,15^.

Metagenomic and transcriptomic approaches have previously been applied to *E. variegatus* and other insect vectors of phytoplasmas, providing valuable insights into their biology and interactions with these plant bacteria^16,17^. However, genome-wide proteomics analyses remain a largely overlooked technique not only in the study of phytoplasma vectors, but more broadly across insect vectors. To date, most proteomic studies on vector-pathogen interactions have focused on blood-feeding insects transmitting human pathogens^18,19^ or plant-infecting viruses^20–23^, with limited attention given to vectors of bacterial plant pathogens^24^. Proteomics provides a robust methodology to comprehensively analyze the functional state of an organism and its molecular responses to environmental stimuli or pathogen challenges. Unlike genomics or transcriptomics, proteomics offers a direct measurement of the effector molecules of biological processes, thereby providing immediate insights into cellular functions and metabolic adaptations.

In this study, we used an integrative approach that combines newly generated genomic data of the *E. variegatus* holobiont (i.e. the insect vector and its associated microbiome) with existing transcriptomic resources to provide the first comprehensive proteomic profile of the holobiont response to FDp acquisition. Specifically, we compared two Liquid Chromatography-Tandem Mass (LC–MS/MS) spectrometry acquisition methods: Data-Dependent Acquisition (DDA) and Data-Independent Acquisition (DIA). DIA was ultimately selected due to its enhanced sensitivity and improved detection of low-abundance peptides, such as those that may originate from the insect microbiome^25^.

## Results

### Construction of a reference proteomic data set for the *E. variegatus* holobiont

As a non-model organism, *Euscelidius variegatus* lacked sufficient genomic data to support the proteomic coverage required by software tools designed for analyzing large mass-spectrometric data sets. To address this limitation, the first step of our study was the construction of a comprehensive reference proteomic database for the *E. variegatus* holobiont. Following the holobiont genome assembly, we successfully reconstructed the complete genomes of its two primary symbionts S*ulcia* and *Nasuia* and the strain of *Sorlinia euscelidii* associated to our lab-reared *E. variegatus* population (Table 1)*.* In addition, we unexpectedly obtained the complete genome of *Pantoea ananatis* (*Pseudomonadota,* hereafter *Pantoea*) (Table 1), a bacterium previously reported to be a component of the microbiome of other insects^26–29^.

**Table 1:** Assembly statistics of the genome of the two primary and two facultative symbionts of *Euscelidius variegatus.*

	*Candidatus* Karelsulcia muelleri	*Candidatus* Nasuia deltocephalinicola	*Pantoea ananatis*	*Sorlinia euscelidii*
Genome length (bp)	188,541	107,039	4,666,554	2,331,141
Genes (total)	226	165	4543	2125
CDSs (total)	191	132	4420	2061
Genes (coding)	189	132	4341	2055
CDSs (with protein)	189	132	4341	2055
Genes (RNA)	35	33	123	64
rRNAs	1, 1, 1 (5S, 16S, 23S)	1, 1 (16S, 23S)	8, 7, 7 (5S, 16S, 23S)	3, 3, 3 (5S, 16S, 23S)
Complete rRNAs	1, 1, 1 (5S, 16S, 23S)	1, 1 (16S, 23S)	8, 7, 7 (5S, 16S, 23S)	3, 3, 3 (5S, 16S, 23S)
tRNAs	30	30	79	51
ncRNAs	2	1	22	4
Pseudo genes (total)	2	0	79	6

bp, base-pair; CDSs, coding sequences; ncRNA, non-coding RNA.

As regards the genomes of *E. variegatus* and the facultative symbiont BEV, the total length of the assembled scaffolds exceeded the estimated size of their respective reference genomes. However, the assembly remained fragmented and did not reach a chromosome-level resolution (Supplementary Information 1). In particular, BUSCO analysis estimated the completeness of the *E. variegatus* genome at 84.5% with respect to the hemipteran core proteome (Table 2). To enhance this completeness, we implemented a stepwise integration of *E. variegatus* transcriptomic data and *Macrosteles quadrilineatus* proteome (NCBI RefSeq assembly: GCF_028750875.1) into our reference database for mass spectrometry analysis, despite potential protein redundancy (Table 2). *Macrosteles quadrilineatus* is the closest phylogenetic species of *E. variegatus* with an available chromosome-level genome assembly. Successive integrations improved the completeness of the core hemipteran gene set, culminating in an almost 100% representation. Proteins with identical amino acid sequences were removed from the dataset.

**Table 2:** Number of core proteins present in our reference proteomic database as assessed by BUSCO using the Hemiptera-specific reference set. BUSCO statistics were calculated under three conditions: (column 1) using the *Euscelidius variegatus* genome alone; (column 2) after adding *E. variegatus* transcriptomic data; and (column 3) after additionally incorporating the *Macrosteles quadrilineatus* proteome. Each column represents a cumulative addition to the reference data used in the previous step, highlighting how successive integration improves completeness of the core hemipteran gene set, culminating in an almost 100% representation.

Total number of core genes used as reference set (Hemiptera)	2510		
	*E. variegatus* genome	+ *E. variegatus* transcriptome	+ *Macrosteles quadrilineatus* proteome
Number (percentage) of complete core genes detected	2121 (84.50%)	2462 (98.09%)	2508 (99.92%)
Number (percentage) of complete and partial core genes detected	2289 (91.20%)	2289 (91.20%)	2509 (99.96%)
Number (percentage) of missing core genes	221 (8.80%)	26 (1.04%)	1 (0.04%)

To achieve a comprehensive representation of the *E. variegatus* holobiont, the reference database used for proteomic profiling (ProteomeXchange dataset identifier: PXD066715) ultimately included: the insect proteome, the predicted proteins from the five newly sequenced bacterial genomes, the polyprotein of Euscelidius variegatus iflavirus 1 (EVV1, GenBank accession: NC_032087), and the predicted proteome of FDp (GenBank accession: CP097583).

### Evaluating the performance of DDA and DIA in proteomic profiling

To compare protein identification across methods, we focused on the number of protein groups, since our aim was to evaluate overall expression rather than specific protein isoforms or post-translational modifications. Additionally, since the insect proteome used in this study comprises predicted proteins from the *E. variegatus* genome, the *E. variegatus* transcriptome and the *M. quadrilineatus* proteome, there is likely to be a high number of protein isoforms. This may limit the identification of unique peptides and consequently affect the reliability of individual protein quantification^30^.

**Fig. 1:**
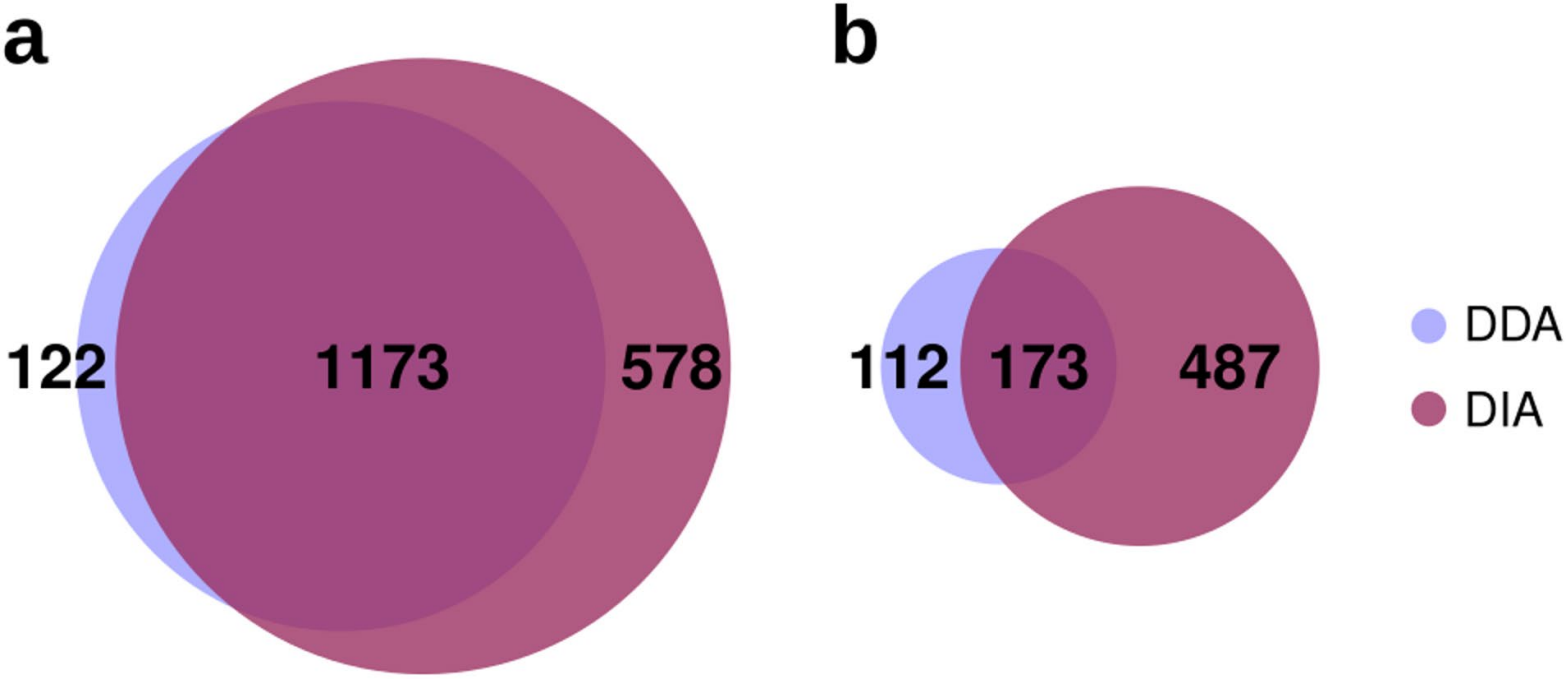
Venn diagrams showing the results of the proteomic analyses conducted using DDA and DIA methods. (**A**) The total number of proteins identified by DDA only, by DIA only, and by both methods. (**B**) The total number of differentially expressed proteins (calculated as the sum of insect- and microbiome-derived proteins) identified by DDA only, by DIA only, and by both methods. 112: 108 insect proteins + 4 microbiome-derived proteins; 173: 168 insect proteins + 5 microbiome-derived proteins; 478: 461 insect protein + 17 microbiome-derived proteins.

Overall, combining all the proteins identified in the eight samples, we identified 1295 distinct protein groups using the DDA approach compared to the 1751 protein groups identified with the DIA approach, representing an increase of 35%. While at least one member of 1173 protein groups was commonly identified by both methods (Fig. 1A), the higher number of protein groups detected exclusively by DIA highlights its superior efficiency and sensitivity in protein identification (ProteomeXchange dataset identifier: PXD066715). The integration of the *Macrosteles* proteome contributed approximately 4% and 16% of the total proteins identified by DDA and DIA, respectively.

Upon further analysis, the DDA approach enabled the identification of an average of 1154 insect proteins in non-exposed samples (H) and 1134 proteins in FDp-exposed samples (FD). The DIA approach facilitated the identification of a higher average number of proteins, with 1579 detected in non-exposed samples (+ 37%) and 1272 in FDp-exposed samples (+ 12%). For microbiome-related proteins, the DDA approach identified an average of 34 proteins in non-exposed samples and 31 proteins in FDp-exposed samples, whereas the DIA approach detected 39 (+ 15%) and 36 (+ 16%) proteins in the respective groups. Notably, in FDp-exposed insects, two phytoplasma membrane proteins (the immunodominant membrane protein—imp and the Variable Membrane Protein A—VmpA) were detected by the DIA approach. Overall, very few proteins were detected from *Sorlinia, Nasuia* and *Pantoea*, whereas BEV and *Sulcia* appeared to be the most metabolically active symbionts, as indicated by the higher number of proteins identified.

### Identification of differentially expressed insect proteins between FDp-exposed and non-exposed insects

DDA analysis identified 276 differentially expressed (DE) insect proteins between FDp-exposed and non-exposed insects, with 78 upregulated and 198 downregulated (false discovery rate [FDR] < 0.05, fold change ≥  ± 0.5; Supplementary Information 2, Fig. 2A). In comparison, the DIA approach identified 638 DE protein, with 64 upregulated and 574 downregulated (false discovery FDR < 0.05, fold change ≥  ± 0.5; Supplementary Information 2, Fig. 2B) representing a 31% increase in the number of DE proteins detected relative to DDA. Of these, 168 DE proteins were commonly identified by both methods (Fig. 1B). Notably, the DIA dataset revealed a considerable number of proteins (137) that were entirely absent in FDp-exposed samples but consistently detected in at least three out of four replicates from non-exposed insects. This threshold (i.e. presence in at least three replicates) was selected based on the expression pattern of two FDp*-*derived proteins, which were consistently detected in at least three replicates of FDp-exposed samples and absent in all non-exposed replicates. This criterion ensures robust identification of condition-specific expression while minimizing false positives.

**Fig. 2:**
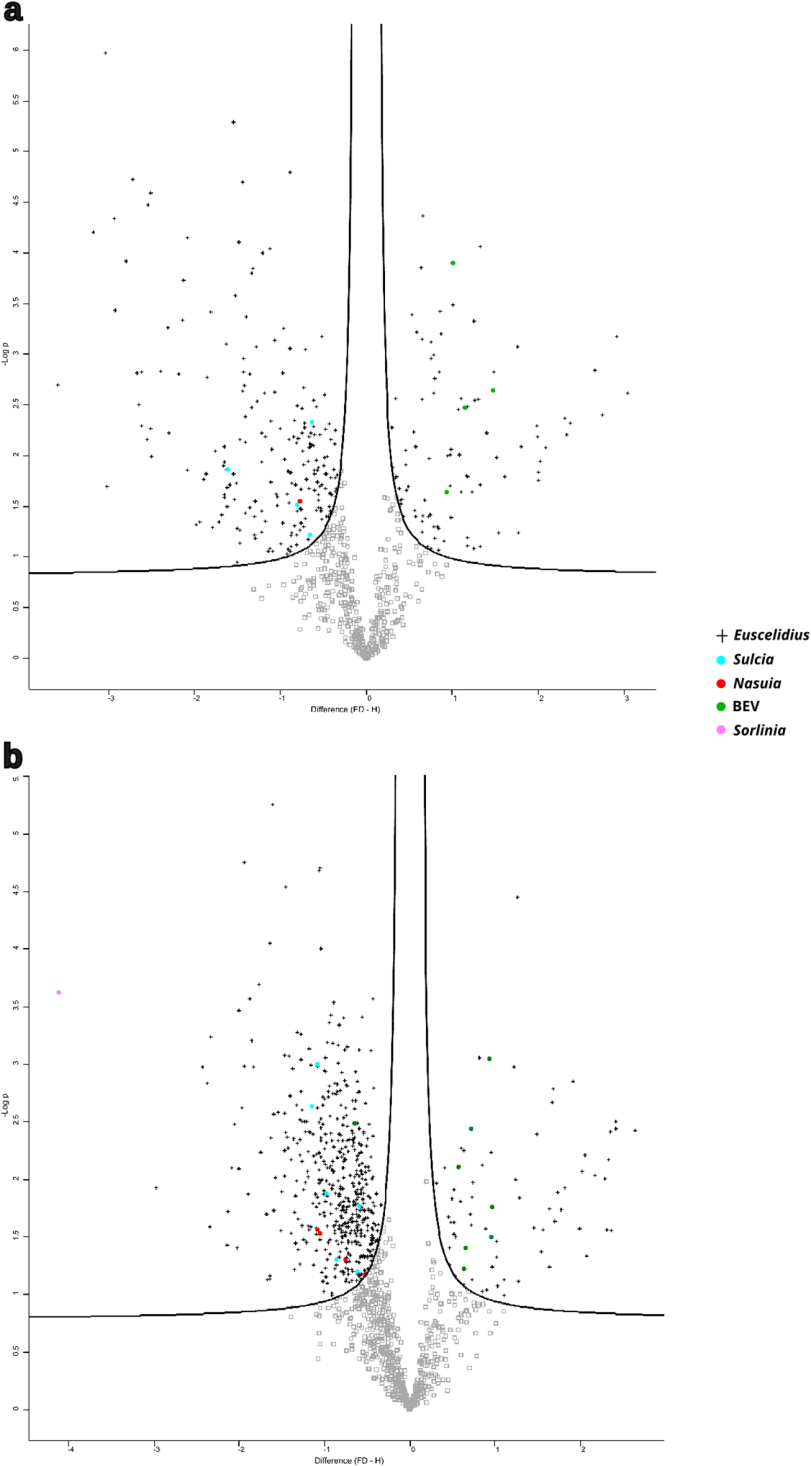
Volcano plots of differentially expressed proteins identified using Perseus. (**A**) Volcano plot of the differentially expressed proteins as identified by Perseus following Data-Dependent Acquisition method. (**B**) Volcano plot of the differentially expressed proteins as identified by Perseus following Data-Independent Acquisition method. Proteins with |log_2_FC|> 0.5 and –log₁₀(p-value) > 1.3 are represented by black crosses (insect), light blue dots (*Sulcia*), greeen dots (BEV), red dots (*Nasuia*) and pink dots (*Sorlinia*). Positive log_2_FC indicates upregulation in FD-exposed samples compared to the control, while a negative log_2_FC indicates downregulation. The x-axis represents the Log₂ fold change (log_2_FC). The y-axis represents the –log₁₀(p-value) obtained using a two-sample t-test with a permutation-based false discovery rate threshold of 5%

**Fig. 3:**
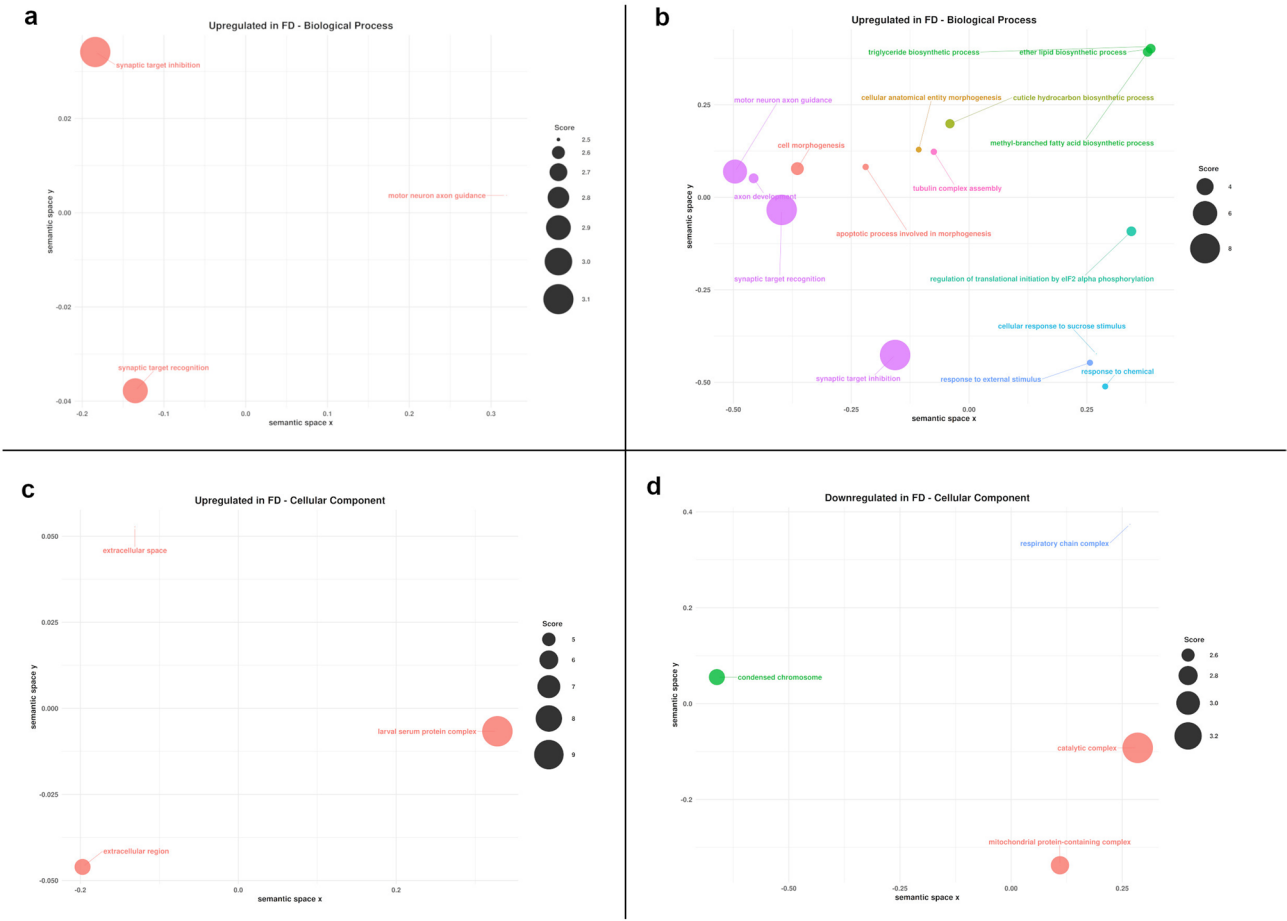
Scatterplots showing semantic similarity of enriched GO terms among differentially expressed insect proteins in response to FDp. (**A**) Enriched Gene Ontology (GO) terms in the Biological Process (BP) ontology for upregulated proteins (DDA); (**B**) Enriched GO terms in the BP ontology for upregulated proteins (DIA); (**C**) Enriched GO terms in the Cellular Component (CC) ontology for upregulated proteins (DIA); (**D**) Enriched GO terms in the CC ontology for downregulated proteins (DIA). GO terms significantly enriched among differentially expressed proteins in FDp-exposed insects (see Supplementary Information 4) were analyzed using the rrvgo R package. Results are divided by GO ontologies: BP, CC and Molecular Function (MF). Redundant GO terms were reduced based on semantic similarity (threshold = 0.7), and the remaining representative terms were visualized in a two-dimensional semantic space. Therefore, the number of GO terms shown in the graph is lower than the one listed in Supplementary Information 4. Point size reflects the score, which is calculated as –log₁₀(q-value). The q-value indicates statistical significance while controlling the False Discovery Rate. GO terms with higher scores are more statistically significant. Only GO terms with a score greater than 2 (i.e. a q-value < 0.01) are shown. Graphs were generated only when more than three enriched terms were identified within a GO category.

To gain functional insights, we analyzed the pathways associated with the DE proteins using the KEGG database (http://www.kegg.jp/kegg/), focusing on BRITE functional hierarchies assigned to proteins upregulated and downregulated in response to FDp infection (Supplementary Information 3). A total of 17 and 29 BRITE functional categories were identified for upregulated and downregulated proteins, respectively, excluding the unclassified proteins. While this classification provided only a broad functional overview of the DE proteins, the results of the GO enrichment analysis (Fig. 3A–D and Supplementary Information 4) highlighted the significantly enriched molecular functions (MF), biological processes (BP), and cellular components (CC) associated to DE proteins. Regarding DE proteins identified by DIA, significantly overrepresented terms were detected in all three GO ontologies for both upregulated and downregulated proteins, with the only exception of the MF ontology among the upregulated proteins, where no significant enrichment was observed (Supplementary Information 4). Although the number of upregulated proteins was approximately nine times lower than that of downregulated proteins, GO enrichment analysis of the biological process ontology identified 27 significantly enriched terms among the upregulated proteins, compared to only three among the downregulated ones. Therefore, it appears that the upregulated proteins were more functionally clustered, leading to their statistical over-representation. In contrast, the downregulated proteins appeared to be spread across a broader range of biological processes, with only a few reaching statistical significance.

DIA results revealed that phytoplasma infection has a pronounced detrimental impact on mitochondrial functions, particularly affecting the respiratory chain complex (Fig. 3D). In contrast, phytoplasma infection led to a statistically significant increment of proteins associated to the extracellular space, i.e. proteins within the cellular matrix or associated to the outer membranes (Fig. 3C). In this regard, it is important to note that some DE predicted proteins that did not show any significant hits (E-value > 0.0001) in the NCBI nr protein database (as indicated by an asterisk in column “best_hit_blastp”, Supplementary Information 2) were predicted to contain a signal peptide (as reported in column “Notes”, Supplementary Information 2).

Both the DDA and DIA approaches consistently demonstrated that pathways associated with neuronal activity were particularly upregulated in FD-exposed insects (Fig. 3A, B). In addition, DIA data specifically revealed an overrepresentation of proteins linked to fatty acid biosynthesis and responses to external stimuli and sucrose (Fig. 3B).

### Identification of differentially expressed microbiome proteins between FDp-exposed and non-exposed insects

Regarding microbiome proteins differentially expressed in response to FDp, the DDA approach identified 9 DE proteins (4 upregulated, 5 downregulated), while the DIA method identified 22 (7 upregulated, 15 downregulated) (Fig. 1B), representing a 44% increase. Of these, five proteins were detected by both methods. Importantly, FDp proteins were excluded from the count of upregulated proteins in FDp-exposed insects to focus solely on microorganisms common to both conditions. Although DIA revealed a higher number of DE microbiome proteins than DDA, the total number remained too low to yield statistically significant results in GO enrichment analysis. Notably, the 15 downregulated proteins largely originated from the two primary endosymbionts of *E. variegatus*, with nine derived from *Sulcia* and four from *Nasuia*. These proteins included three chaperonins and several key enzymes involved in amino acid biosynthesis, specifically within the valine, leucine, and isoleucine biosynthesis pathway, as well as the phenylalanine, tyrosine, and tryptophan biosynthesis pathway. In contrast, all upregulated microbiome-derived proteins were associated with the secondary endosymbiont BEV. Notably, among the upregulated proteins was a major capsid protein (GenBank Accession: QVJ82353.1), previously identified as part of a BEV temperate phage^31^. Additionally, three membrane proteins were identified: OmpA porin, involved in cell envelope stability, adhesion, invasion, biofilm formation, and phage recognition; KdgM, an oligogalacturonate-specific porin and a subunit of the ribose ABC transporter.

## Discussion

Recent advances in instrumentation and strategies for data acquisition in mass spectrometry have significantly enhanced the levels of accuracy and sensitivity of proteomics so that it can now provide a more detailed and dynamic description of biological systems compared to transcriptomics, particularly by capturing the post-transcriptional and translational regulations of gene expression. However, a fundamental constraint of proteomic analyses is their reliance on comprehensive reference databases, a limitation that is particularly pronounced when working with non-model organisms. The initial step of our study was the creation of the most extensive possible database by sequencing the genome of the *E. variegatus* holobiont and predicting all its putative protein-coding genes.

The cumulative length of the obtained genomic scaffolds for *E. variegatus* should cover approximately the expected genome size of around 1.3 Gb, as estimated based on the chromosome-level genome assembly of the closest phylogenetically related insect, *M. quadrilineatus*^32^. Despite that, BUSCO analysis demonstrated that *E. variegatus* genome only encompasses 84.5% of the expected core proteome of hemipterans. Consequently, some scaffolds are likely to be redundant. This redundancy likely persisted because bioinformatic algorithms were unable to merge them, probably due to the large quantity of repetitive elements (e.g., tandem repeats and interspersed repeats) that have been previously observed in other leafhopper genomes^32–34^.

Due to the current incompleteness of the *E. variegatus* genome, integrating genome-derived proteins with *Euscelidius* transcriptomic-derived proteins and proteomic information from *M. quadrilineatus* was essential for improving downstream protein identification. This approach is consistent with previous studies that have emphasized the difficulties associated with proteomics in non-model organisms with limited genomic resources^35–37^. Although BUSCO analysis indicated that the *Macrosteles* proteome contributed only around 2% to the completeness of *E. variegatus* core proteome, it accounted for between 4 and 16% of the total identified protein groups, depending on the acquisition method used. This discrepancy likely reflects the higher number and accuracy of protein predictions derived from the chromosome-level genome assembly of *Macrosteles*, which includes not only core proteins, but also *Cicadellidae*/*Deltocephalinae-*specific proteins and isoforms. Consequently, certain conserved proteins, along with some missing *Euscelidius* proteins, are likely to be more accurately predicted and identified in the *Macrosteles* proteomic database than in the *E. variegatus* one. Additionally, the DIA approach resulted in the identification of a greater number of differentially expressed *Macrosteles* proteins, as well as more proteins in general, when compared to the DDA approach. While effective for identifying abundant proteins, DDA is frequently subject to limitations such as reduced reproducibility and suboptimal coverage of lower-abundance proteins. Conversely, DIA generates a comprehensive, high-resolution dataset that includes virtually all detectable peptides. It facilitates the identification of differentially expressed proteins, including the less abundant ones, such as those derived from the insect microbiome. Such particular achievement was supported by the reconstruction of complete, high-quality genomes of the two primary endosymbionts (*Sulcia* and *Nasuia*), two associated bacteria (*S. euscelidii* and *P. ananatis*), and a partial genome of BEV.

In view of the aforementioned advantages, DIA-based results were considered more comprehensive than those derived from DDA to provide a proteomic foundation for exploring the response of the insect and its microbiome to the challenge posed by the phytoplasma.

Notably, the presence of the plant pathogen in FDp-exposed insects was confirmed by the identification of two FDp surface-exposed proteins: VmpA and imp. VmpA facilitates the phytoplasma adhesion to specific molecules on the surface of insect midgut cells^38^, while Imp is involved in interactions between FDp and insect gut proteins^39,40^.

Overall, the presence of phytoplasma resulted in a greater number of proteins being downregulated than upregulated, indicating a general suppression of protein expression compared to control conditions. This observation aligns with previous findings reporting that FDp has detrimental effects on the survival and fecundity of its natural insect vector, *S. titanus*^41^. Similarly, infection with certain viruses, such as baculoviruses, can lead to a shutdown of protein synthesis in insect cells^42^. This strategy is employed by viruses to suppress the host’s immune response and create an environment that is more favorable for their own replication. In particular, according to the GO enrichment analysis conducted on DIA data*, E. variegatus* responded to FDp with a downregulation of proteins associated with the mitochondrial compartment, including key enzymes involved in the respiratory electron transport chain. This pattern suggests a potential impairment of mitochondrial energy metabolism in response to infection. Indeed, previous RNAi experiments have shown that RNAi-mediated silencing of ATP synthase β in *E. variegatus* leads to a significant reduction in phytoplasma multiplication, likely due to ATP limitation^43^. It is plausible that the downregulation of respiratory chain components and mitochondrial functions observed in phytoplasma-infected insects may represent an insect defense mechanism aimed at limiting phytoplasma proliferation. Additionally, in *Euscelidius variegatus*, the Major Antigenic Membrane Protein (Amp) of *“Candidatus* Phytoplasma asteris” has been shown to selectively interact with the beta subunit of the mitochondrial ATP synthase^44^, suggesting that phytoplasma may exploit host cell energy metabolism during colonization of the insect vector. No data is present in literature about the interaction of phytoplasma and insect mitochondria; however, in plants this association has been observed occasionally. In *Arabidopsis thaliana* infected with FDp, phytoplasma cells were found near mitochondria, particularly in root tissues^45^; similarly, in the phloem of tomato plants infected with the Columbia Basin PPT phytoplasma, bacterial cells were present at the contact site between the endoplasmic reticulum and mitochondria^46^. Although this association has not been studied across other phytoplasma–plant-vector interactions, it raises the possibility that phytoplasmas may similarly rely on host mitochondria as an external source of ATP. This is consistent with genomic evidence indicating that phytoplasmas lack key components of the ATP synthesis machinery^47^. The potential recruitment or manipulation of mitochondria by phytoplasmas may therefore serve a dual function: not only to access host-derived energy, but also as a strategy to interfere with mitochondrial-mediated defense signaling. Together, these observations point to a conserved and potentially critical role of host mitochondria in phytoplasma-host interactions across both plant and insect systems, warranting further investigation into mitochondria as a shared interface exploited by phytoplasmas for survival and propagation.

GO enrichment analysis also showed that axogenesis, lipid biosynthesis and response to external stimuli were overrepresented among the upregulated proteins in *E.* v*ariegatus* following exposure to FDp. We can speculate that the presence of phytoplasma induces changes in insect behaviour, such as increased movement or altered feeding habits, as demonstrated in other insect vectors^48,49^. The enrichment of axogenesis-related proteins suggests possible remodeling or development of neural circuits, which could affect sensory perception or motor function in response to external stimuli. Additionally, the upregulation of lipid biosynthesis pathways, particularly ether lipids and triacylglycerols (TAGs), could impact various physiological functions, including changes in membrane composition that may be required to accommodate the phytoplasma. In addition to their structural and energetic roles, lipids play crucial functions in antioxidant defense, cell signaling, immune regulation, and synaptic activity within the nervous system^50^.

Arylphorins (a major class of hexamerins) and other hexamerins form the larval serum protein complex (GO: Cellular Component), a large multimeric storage complex that functions as an energy and amino-acid reserve in insects^51^. The increased abundance of these proteins upon FDp exposure may arise from two processes: (i) a compensatory response to phytoplasma-driven depletion of nutrient reserves normally stored in hexamerins, and/or (ii) their involvement in the humoral immune response to control the phytoplasma, similarly to what has been described for another leafhopper, *Circulifer haematoceps*, in response to the plant-pathogenic bacterium *Spiroplasma citri*^52^.

**Fig. 4:**
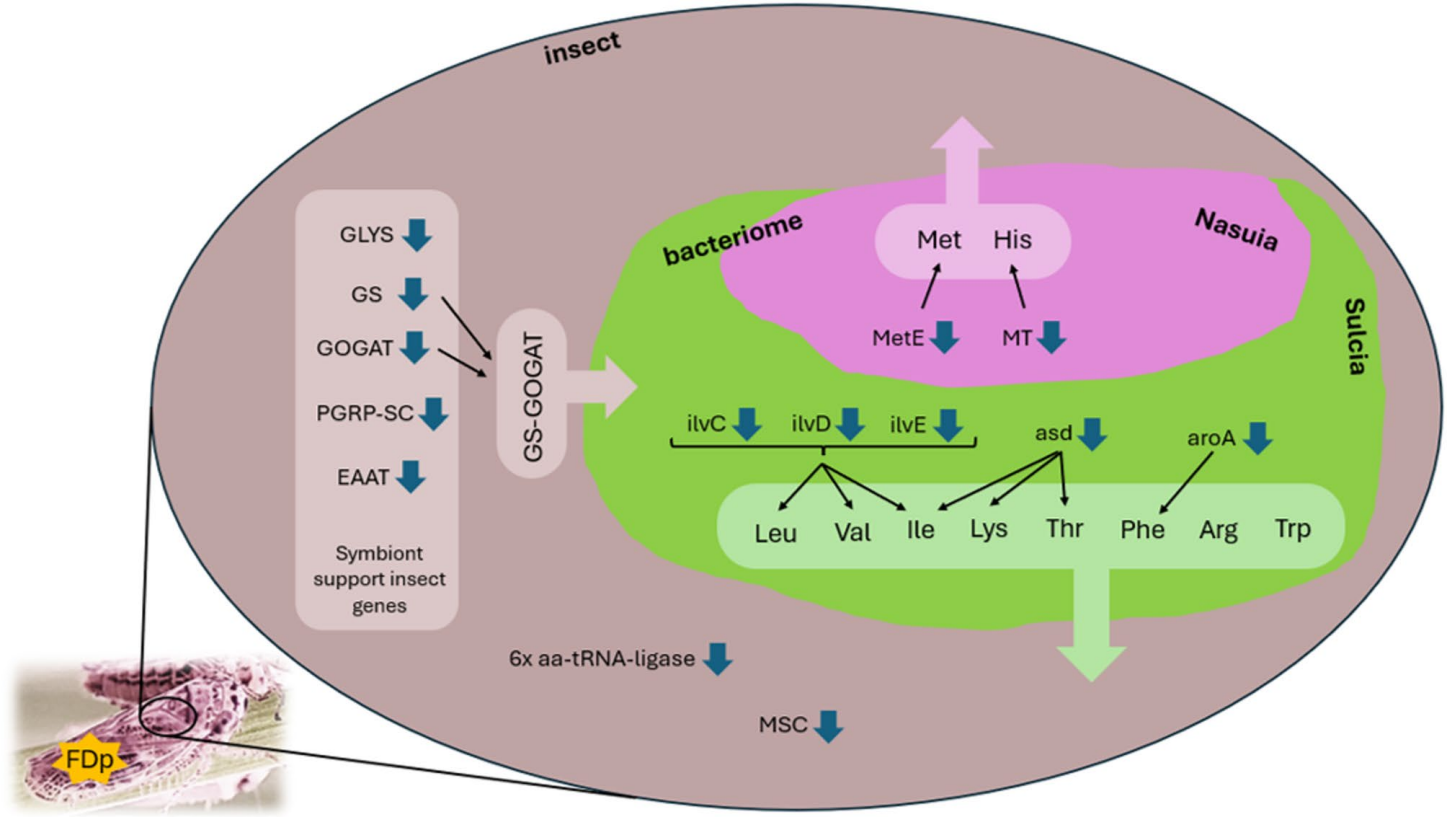
Effect of *Euscelidius variegatus* exposure to FDp on protein involved in metabolic cooperation between the insect host and its primary endosymbionts. Brown area represents insect tissues, pink/green area represents a bacteriome with bacteriocytes hosting *Nasuia* (pink sector) and *Sulcia* (green sector) (Adapted from Fig. 2 in Bennet and Moran, 2013). Blue arrows indicate downregulation; black arrows indicate involvement in biosynthesis. Insect down-regulated proteins belonging to the “symbiont support genes set” as defined by Vasquez et al. 2024^32^: glycine-tRNA ligase (GLYS), glutamine synthetase 2 cytoplasmic (GS), glutamate synthase large chain (GOGAT), peptidoglycan recognition protein 3-like (PGRP-SC) and excitatory amino acid transporter-like (EAAT); aa-tRNA-ligase: insect down-regulated tRNA ligases; MSC: insect down-regulated multi-tRNA synthetase complex. *Nasuia* down-regulated proteins involved in aminoacid biosynthesis: 5-methyltetrahydropteroyltriglutamate-homocysteine (MetE), S-methyltransferase HisA/HisF-related TIM barrel protein (MT). *Sulcia* down-regulated proteins involved in aminoacid biosynthesis: ketol-acid reductoisomerase (ilvC), dihydroxy-acid dehydratase (ilvD), branched-chain amino acid aminotransferase 2 (ilvE), aspartate-semialdehyde dehydrogenase (asd), 3-deoxy-7-phosphoheptulonate synthase/chorismate mutase (aroA). GS-GOGAT: glutamine synthetase/glutamate synthase cycle.

Interestingly, five *E. variegatus* homologues of the 20 genes identified by Vasquez et al.^32^ in *M. quadrilineatus* as putative symbiont-support genes were found to be downregulated in response to FDp (see column “Notes” in Supplementary Information 2). *Macrosteles quadrilineatus* harbors the same two obligate endosymbionts as *E. variegatus* and these 20 insect genes, predicted to be under positive selection, were identified among those likely to compensate for the incomplete metabolic functions of the highly degraded symbiont genomes. The five *E. variegatus* downregulated proteins are glycine-tRNA ligase, glutamine synthetase 2 cytoplasmic, glutamate synthase large chain (i.e. Uncharacterized protein LOC128983935), peptidoglycan recognition protein 3-like and excitatory amino acid transporter-like. In particular, it has been found in other hemipterans that both symbionts have lost the ability to produce and transport glutamine and glutamate, which are required to synthesize essential amino acids for their insect hosts^53^. Therefore, the activity of the insect GS/GOGAT (glutamine synthetase/glutamate synthase) pathway is essential for the survival of the entire holobiont. In addition to glycine-tRNA ligase, six other tRNA ligases and a component of the multi-tRNA synthetase complex were downregulated upon FDp challenge, indicating once more a decrease in the overall rate of protein production (Fig. 4).

From the perspective of the two primary symbionts, all differentially expressed proteins encoded by *Sulcia* and *Nasuia* were found to be downregulated in response to FDp infection. Such reduction of protein expression could result either from true protein downregulation or from a decrease in the number of bacterial cells present within the insect host. A global decline in bacterial cell number would be expected to reduce all identified bacterial proteins; however, only nine out of the 18 *Sulcia* proteins identified by DIA showed decreased levels. This pattern, therefore, suggests that specific protein downregulation, rather than a general reduction in bacterial titer, is the more likely explanation.

KEGG pathway analysis revealed that three out of the nine down-regulated *Sulcia* proteins (ketol-acid reductoisomerase, dihydroxy-acid dehydratase and branched-chain amino acid aminotransferase 2) are involved in the biosynthesis of the branched-chain amino acids valine, leucine, and isoleucine. Notably, isoleucine, leucine and valine are among the eight essential amino acids provided by *Sulcia* to the insect host, alongside arginine, phenylalanine, tryptophan, lysine, and threonine. Two further *Sulcia* downregulated proteins are involved in the synthesis of amino acid precursors: the bifunctional enzyme 3-deoxy-7-phosphoheptulonate synthase/chorismate mutase (DAHPS/CM) type II, which contributes to phenylalanine biosynthesis, and the aspartate-semialdehyde dehydrogenase, which is involved in the synthesis of lysine, threonine, and isoleucine (Fig. 4).

*Nasuia*, which complements *Sulcia* by providing the insect with methionine and histidine, showed four downregulated proteins, which included precisely two enzymes involved in the biosynthesis of methionine and histidine: 5-methyltetrahydropteroyltriglutamate–homocysteine and S-methyltransferase HisA/HisF-related TIM barrel protein, respectively (Fig. 4).

Overall, these data suggest that FDp has a negative impact on the reciprocal metabolic support between the host insect and its primary endosymbionts, consequently resulting in an imbalance in amino acid synthesis and exchange. In this scenario, we observed an increase in the expression of some BEV membrane proteins involved in nutrient uptake, possibly reflecting an opportunistic adaptation of BEV to the phytoplasma-induced changes in host physiology. When it was first characterized in 1987, BEV showed, in fact, a certain degree of pathogenicity towards *E. variegatus*^54^

## Conclusions

Recent advances in microbial ecology have highlighted the profound influence of insect-associated microbiomes, including bacteria, fungi, and viruses on host physiology, immunity, vector competence, and overall host fitness^6^. The identification of proteins from the primary symbionts, both localized in bacteriomes, as well as from BEV, predominantly found in gut and haemocoel, demonstrates that protein extraction from whole insect bodies allows also the detection of microbial proteins without the need for tissue dissection. However, targeted dissection of specific organs could increase the number and diversity of microbial proteins detected, thereby providing a more detailed understanding of the metabolic exchanges between the insect and its microbiome.

Proteins that are upregulated in response to FDp might represent promising targets for rational drug design (e.g., small molecules, peptides, or antibodies) or for gene silencing via dsRNA-mediated RNA interference. Their upregulation might be either a consequence of the phytoplasma manipulation of the insect metabolism or an active defense mechanism developed by the insect against the phytoplasma multiplication. For example, the silencing of natterin-1 in E. variegatus, which was identified as overexpressed in this study upon FDp exposure (Supplementary Information 2), was previously shown to impair phytoplasma multiplication^40^.

While examples of targeted drug development exist for insect vectors of other pathogens, such as the use of mycophenolic acid to inhibit Dengue and Zika virus infections by targeting the mosquito inosine monophosphate dehydrogenase^55,56^, no such strategies have yet been developed against vectors of phytoplasmas.

The remarkable potential of genome-wide proteomic analysis in insect pests does lie in the possibility to uncover new species-specific molecules or pathways that may be targeted to disrupt essential biological processes with high precision and minimal off-target effects, including those involved in symbiotic relationships with their microbiome.

## Methods

### Plants, insects, and phytoplasma strain

Oat (*Avena sativa*) and broad bean (*Vicia faba* var. “Agua-dulce Supersimonia”) plants were grown from seed in pots in a greenhouse and maintained at 24 ± 2 °C. Two weeks after sowing, *A. sativa* plants were used to rear phytoplasma-free colonies of the leafhopper *Euscelidius variegatus* and *V. faba* plants were used as host plants to maintain isolates of the FDp. *Euscelidius variegatus* was originally collected in the Piedmont region (Italy) and has been continuously reared in plastic and nylon cages inside growth chambers set at 20–25 °C with a 16:8 light–dark photoperiod. The FDp strain FD-D^57^, also originally isolated in Piedmont, is routinely maintained on *V. faba* through sequential transmission by *E. variegatus*^16^. For phytoplasma acquisition, fifth-instar nymphs from phytoplasma-free colonies were fed on FDp-infected broad beans for an acquisition access period (AAP) of 7 days and then transferred to oats for a 28-day latency period (LP). At 35 days post-acquisition, the surviving insects were analyzed for phytoplasma infection (as described in Rossi et al. 2023^58^) and used in experiments.

### DNA extraction and sequencing

High molecular weight genomic DNA was extracted from a pool of 30 ethanol surface-sterilized *E. variegatus* individuals using the cetyltrimethylammonium bromide (CTAB) protocol^59^. A total of 6 μg of DNA was obtained, and its quality and quantity were assessed using a Qubit fluorometer (Thermo Fisher Scientific, USA) and agarose gel electrophoresis.

DNA was submitted to Novogene Co., Ltd. (Beijing, China) for library preparation and sequencing. A SMRTbell library was constructed and sequenced on the PacBio Sequel IIe platform (Pacific Biosciences, CA, USA), generating approximately 100 Gb of raw long-read data. The same DNA sample was also used to prepare a short-read paired-end library (insert size ~ 350 bp), which was sequenced on the Illumina NovaSeq 6000 platform, producing 140 Gb of raw data with 2 × 250 bp reads.

### Genome assembly

To limit memory usage, random subsets corresponding to approximately one-third of the total PacBio long-read data (~ 31 Gb) were generated using seqtk v. 1.4-r122 (https://github.com/lh3/seqtk). The draft assemblies were produced using Flye v. 2.9.1-b1780^60^ and polished using Illumina short-read data. Illumina reads were first subjected to quality control using the BBTools suite (BBMap—Bushnell B.—sourceforge.net/projects/bbmap/) to remove adapter sequences, low-quality bases, and potential contaminants. Subsequently, reads were aligned to the Flye assemblies using BWA-MEM v. 0.7.15-r1140 (Liet al. 2009), and polishing was performed with Pilon v. 1.24^61^ through three iterative rounds to correct residual base errors and indels.

As the sequenced sample was a mixture of the host and bacterial symbionts, the initial assembled contigs were identified using BLASTN searches against the NCBI nt database, separated into bacterial and insect sequences, and, within each group, assemblies were merged using Quickmerge (https://github.com/mahulchak/quickmerge) to improve contiguity. This separation was guided by the existing knowledge that *E. variegatus* harbors two primary bacterial symbionts, *Sulcia* and *Nasuia*, as well as two facultative symbionts, BEV and *S. euscelidii,* whose genome has been recently sequenced (BioProject: PRJNA1026792). Assembly quality was assessed using QUAST v. 5.3.0^62^. *Euscelidius variegatus* genome completeness was assessed using BUSCO v5 with the *hemiptera_v10* ortholog set.

### Gene prediction

Prediction of insect coding sequencing and proteins was performed using the MAKER annotation pipeline v. 2.31.11^63^ for eukaryotic organisms. The draft assembled genome and transcript evidence from a previous *E. variegatus* RNA-seq project (Bioproject PRJNA393620) were used as input. Repeat masking was performed within the MAKER pipeline using RepeatMasker, which was configured to use the Dfam database (bundled with MAKER) as the primary source for transposable element profiles. MAKER was used in two iterative rounds. In the first round, insect transcripts were aligned to the genome to generate initial gene predictions. In the second round, these predictions were refined using AUGUSTUS v. 3.4.0 with *Acyrthosiphon pisum* gene models and a high-confidence subset of gene models derived from the first round. Final gene models were extracted using gffread and used to train a species-specific AUGUSTUS ab initio predictor. This trained model was then employed in a final MAKER run to generate the definitive set of coding sequences and predicted proteins.

Scaffolds identified as bacterial were annotated for protein-coding genes using PROKKA v. 1.14.6^64^.

### Functional annotation of the assembled genomes

Functional annotation of all predicted protein-coding sequences was performed using blastp analysis against the NCBI nr database (threshold: Evalue < 0.0001) and eggNOG-mapper v2 (http://eggnog-mapper.embl.de/), based on orthology assignments from the eggNOG 5.0 database. In addition to orthologous group assignments, eggNOG-mapper provides functional annotations from multiple sources, including Gene Ontology (GO) terms and the BRITE hierarchy, which were used for downstream analyses. GO annotations characterized predicted gene products based on their putative biological roles, molecular functions, and subcellular localizations. BRITE codes, derived from the KEGG database, were used to assign high-level functional categories to the identified proteins. Functional pathway assignments were specifically derived from the KEGG reference pathways of the hemipteran insect *Halyomorpha halys* (hhal).

Proteins without a significant hit in the NCBI nr database were further analyzed with SignalP 5.0^65^ to provide additional functional insights regarding their potential secretion (probability threshold > 0.5).

### Protein extraction and quantification

Total proteins were extracted from insects either exposed (FD) or non-exposed (H) to FDp. Four biological replicates were analyzed for each condition, resulting in a total of 200 insects per treatment. For each replicate, 50 insects were homogenized in a mortar using liquid nitrogen. Once the insects were ground into a fine powder, 1 mL of RX buffer (0.1% Triton X-100, 100 mM KCl, 3 mM NaCl, 3.5 mM MgCl2, 1.25 mM EGTA, and 10 mM Hepes, pH 7.3)^47^ was added. The samples were then sonicated for 1 min at room temperature and centrifuged at 13,000 rpm for 5 min. The supernatant was collected and precipitated with methanol/chloroform/water, as described by Wessel and Flügge (1984). The samples were quantified by means of the 2-D Quant Kit (Cytiva) with an adapted procedure for the 96-well microplate reader Tecan (Infinite M Nano). An aliquot of 100 μg of proteins was resuspended in 100 μL of 25 mM ammonium bicarbonate. The samples were reduced with dithiothreitol (DTT) to the final concentration of 20 mM, kept for 1 h at 37 °C, and alkylated with iodoacetamide (IAA) at the final concentration of 55 mM, for 1 h in the dark at room temperature (RT). The residual IAA was quenched in a second incubation step with DTT at the final concentration of 30 mM for 15 min at RT. The protein mixtures were digested O/N at 37 °C under shaking with modified porcine proteomic grade trypsin (1 μg/μL) at a 1:20 trypsin-protein ratio. An aliquot of 5 μL of pure formic acid (FA) was added to stop the digestion. The tryptic peptide mixtures were desalted using Strata C18-E SPE tubes. Briefly, the tubes were conditioned with 3 mL of methanol, then equilibrated with 3 mL of MilliQ water. The samples, previously diluted with 2 mL of 0.1% FA, were loaded in the Strata-X tubes. The tubes were then washed with 1 mL of 5% methanol, and the peptides were eluted with 3 mL of methanol. After desalting, the samples were dried in a 5301 Eppendorf Concentrator (Eppendorf, Hamburg, Germany) and suspended in 50 μL of 0.1% (v/v) FA/2% acetonitrile (ACN) before the analysis.

### UHPLC-HRMS data acquisition: DDA and DIA methods

The high-resolution mass spectrometry (HRMS) analyses were performed using an Orbitrap Q Exactive Plus, coupled to an ultra-high-performance liquid chromatography (UHPLC) binary pump system (Vanquish Thermo Fisher Scientific, Waltham, Massachusetts, USA) with a HESI probe.

The stationary phase was a Luna C18 HPLC Column (150 × 1 mm, 3 µm; Phenomenex, Castelmaggiore, Italy). The oven temperature was set at 55 °C, and the autosampler was set at 4 °C. The mobile phases were 0.1% (v/v) FA in MilliQ water (A) and 0.1% (v/v) FA in 30% ACN 70% MetOH (B), and they were eluted at a flowrate of 70.0 μL/min, with an increasing concentration of solvent B, from 3 to 50%, over 80 min and with 70% in 5 min, and 100% in 1 min, for 5 min, and after reconditioned at 3% of B for 15 min. The injection volume was 4.0 μL.

The DDA method was in positive polarity mode, spray voltage of 3500 V, Sheat Gas 35, Aux Gas 10, probe heater 330 °C and S-Lens RF Level 60. Mass spectra were acquired in Full MS-ddMS2 mode. The instrument was set up so that Full MS spectra were acquired in an m/z scan range of 250–1500, the resolution was set at 70,000, the maximum IT was 200 ms, the AGC target was 3 × 10^6^. Up to 6 of the most intense ions in MS1 were selected for fragmentation in the MS/MS mode. The fragmentation spectra resolution was set at 17,500 for the MS/MS spectra, with a dynamic exclusion of 10 s and an isolation window of 2.0 m/z. The normalized collision energy was set at 28, the maximum IT at 110 ms and the AGC target at 1 × 10^5^.

The DIA method was composed of 20 consecutive MS2 windows acquired at 17,500 resolution, with an AGC target of 1 × 10^6^ and a maximum injection time of 60 ms. In details the DIA method enclosed 4 windows with an isolation window of 31 m/z, 13 windows with an isolation of 21 m/z (AGC target of 2 × 10^5^), and 3 windows with an isolation window of 51 m/z; the overlap for each window was equal to 0.5 m/z. The resulting m/z range was from 370 to 900.

### Analysis parameters for DDA and DIA proteomic data

The Data-Dependent Acquisition (DDA) raw files were analyzed using MaxQuant (version 2.4.2.0), with peptide identification performed against a custom-made predicted proteome of the holobiont *E. variegatus.* The search was performed with trypsin specificity, allowing up to two missed cleavages. Carbamidomethylation of cysteine was set as a fixed modification, while oxidation of methionine and N-terminal acetylation was set as variable modifications. A false discovery rate (FDR) of 1% was applied at both the peptide-spectrum match (PSM) and protein levels. Label-free quantification (LFQ) was enabled, with a minimum ratio count of 2, and the ‘match between runs’ feature was activated.

The Data-Independent Acquisition (DIA) raw files were analyzed using DIA-NN (version 1.9.1) in library-free mode. In silico spectral libraries were generated from the same reference proteomes used in the MaxQuant analysis. An FDR of 1% was applied at both the precursor and the protein group levels. Protein quantification was performed with the following parameters enabled: trypsin was specified as the digestion enzyme, allowing for up to two missed cleavage site; charge range of 1–4 for peptides with 7–30 amino acids was set; peptidoform-level quantification, match-between-runs (MBR), heuristic protein inference, no shared spectra, single-pass mode, Robust LC (high precision), and retention time (RT)-dependent quantification mode were selected. The digestion settings and the allowed protein modifications in DIA-NN were configured to match those used in the MaxQuant analysis.

### Statistical analysis of differentially expressed proteins

Proteomic quantification results from MaxQuant and DIA-NN were analyzed using Perseus (version 1.6.15.0). Label-free quantification (LFQ) intensities from MaxQuant and protein intensity values from DIA-NN were log₂-transformed and normalized by median subtraction across columns. For differential expression analysis, only proteins quantified in at least three biological replicates per condition were retained. Differential expression analysis was performed using a two-sample *t*-test with a permutation-based false discovery rate (FDR) threshold of 5%. Proteins that were not detected in any replicate of one condition but were consistently detected in at least three replicates of the other condition were not included in the statistical analysis due to the absence of variance estimates. However, they were retained and reported as condition-specific proteins and considered overexpressed in the condition where they were detected.

Gene Ontology enrichment analysis was conducted using the GOEnrichment tool (https://github.com/DanFaria/GOEnrichment) with the GO file go-basic.obo. For each proteomic approach (DIA and DDA), the combined set of all proteins identified in non-exposed samples was used as the annotation (population) set, and the corresponding lists of differentially expressed proteins after the FDp challenge served as the study set. Multiple testing correction was applied using the Benjamini–Hochberg method, with a significance threshold set at *p* < 0.01. To reduce redundancy and improve visualization, the results were further refined using the rrvgo R package v.1.20.0. The terms with semantic similarity below 0.7 were grouped, and the org.Dm.eg.db annotation package (*Drosophila melanogaster* library) was used for functional annotation and graphical representation. REVIGO summarizes and visualizes GO terms by identifying a representative subset based on semantic similarity, when at least three terms are available within the same GO category.

## Supplementary Information

Below is the link to the electronic supplementary material.

Supplementary Material 1.

Supplementary Material 2.

Supplementary Material 3.

Supplementary Material 4.

## Data Availability

All the assembled genomes are available at the NCBI GenBank database under the BioProject accession number PRJNA1057536. The mass spectrometry proteomics data have been deposited to the ProteomeXchange Consortium via the PRIDE partner repository with the dataset identifier PXD066715 and [10.6019/PXD066715] (https:/doi.org/10.6019/PXD066715) Token: WByXJicLDy5A. This dataset comprises the reference proteomic database that was used for all the proteomic analyses and the raw output files generated by MaxQuant and DIA-NN. To maintain consistency between the protein database and the deposited output files of DDA and DIA analyses, in those files *Sorlinia* proteins keep the original prefix ‘asaia’, because *Asaia* sp. was the closest phylogenetic match to the bacterium identified in the *E. variegatus* microbiome before the releasing of *S. euscelidii* genome. The other prefixes are the same as the ones mentioned before.
