# Supplementary material for: Rewiring the proteome of the Euscelidius variegatus holobiont in response to Flavescence dorée phytoplasma
Source: Sci Rep. 2025 Dec 4;16:1171. doi: 10.1038/s41598-025-30920-7 (PMC12789426; doi:10.1038/s41598-025-30920-7)
Supplement: Supplementary file 1 — Supplementary Material 1. [file 41598_2025_30920_MOESM1_ESM.pdf]

Rewiring the proteome of the *Euscelidius variegatus* holobiont in response to *Flavescence dorée* phytoplasma

Simona Abbà[1*], Marta Vallino[1], Simona Cirrincione[2], Cristina Lamberti[2], Beatrice Aiuto[2], Francesco Romaniello[3], Luciana Galetto[1], Cristina Marzachì[1], Domenico Bosco[4] & Marika Rossi[1]

Supplementary Information 1

Genome assembly statistics of the contig of the insect *E. variegatus* and its facultative symbiont BEV.

N50: sequence length of the shortest contig such that 50% of the entire assembly is contained in contigs equal to or larger than this value; NG50: the same as N50 except that it is 50% of the known or estimated genome size; N90: sequence length of the shortest contig such that 90% of the entire assembly is contained in contigs equal to or larger than this value; NG90: the same as N90 except that it is 90% of the known or estimated genome size; L50: count of the smallest number of contigs whose length sum makes up half of the entire assembly; LG50: the same as L50 except that it is referred to the known or estimated genome size; L90: count of the smallest number of contigs whose length sum makes up 90% of the entire assembly; LG90: the same as L90 except that it is referred to the known or estimated genome size.

	Euscelidius variegatus	**BEV**
Total length (bp)	1,532,370,202	5,140,771
# contigs	50,182	6
Largest contig (bp)	593,122	4,353,119
Estimated reference length (bp)	*Macrosteles quadrilineatus* (GenBank: GCF_028750875.1) 1,300,000,000	*Symbiopectobacterium purcellii* (GenBank: NZ_CP081864) 4,942,431
GC (%)	34.88	52.17
N50	41,798	4,353,119
NG50	54,340	4,353,119
N90	14,096	395,570
NG90	25,237	395,570
L50	10,087	1
LG50	6,581	1
L90	35,570	2
LG90	19,824	2
