# Supplementary figures and images for: Rewiring the proteome of the Euscelidius variegatus holobiont in response to Flavescence dorée phytoplasma
Source: Sci Rep. 2025 Dec 4;16:1171. doi: 10.1038/s41598-025-30920-7 (PMC12789426; doi:10.1038/s41598-025-30920-7)

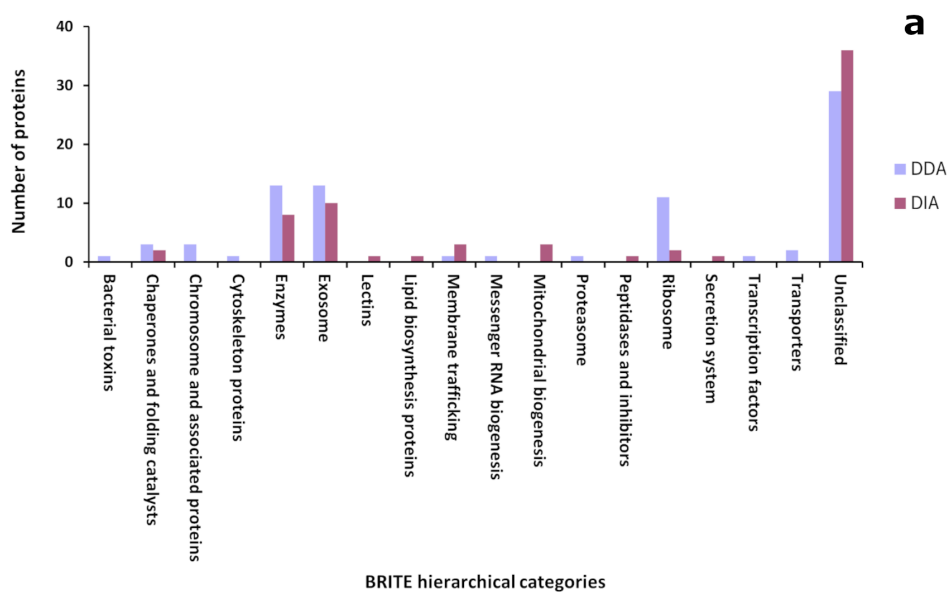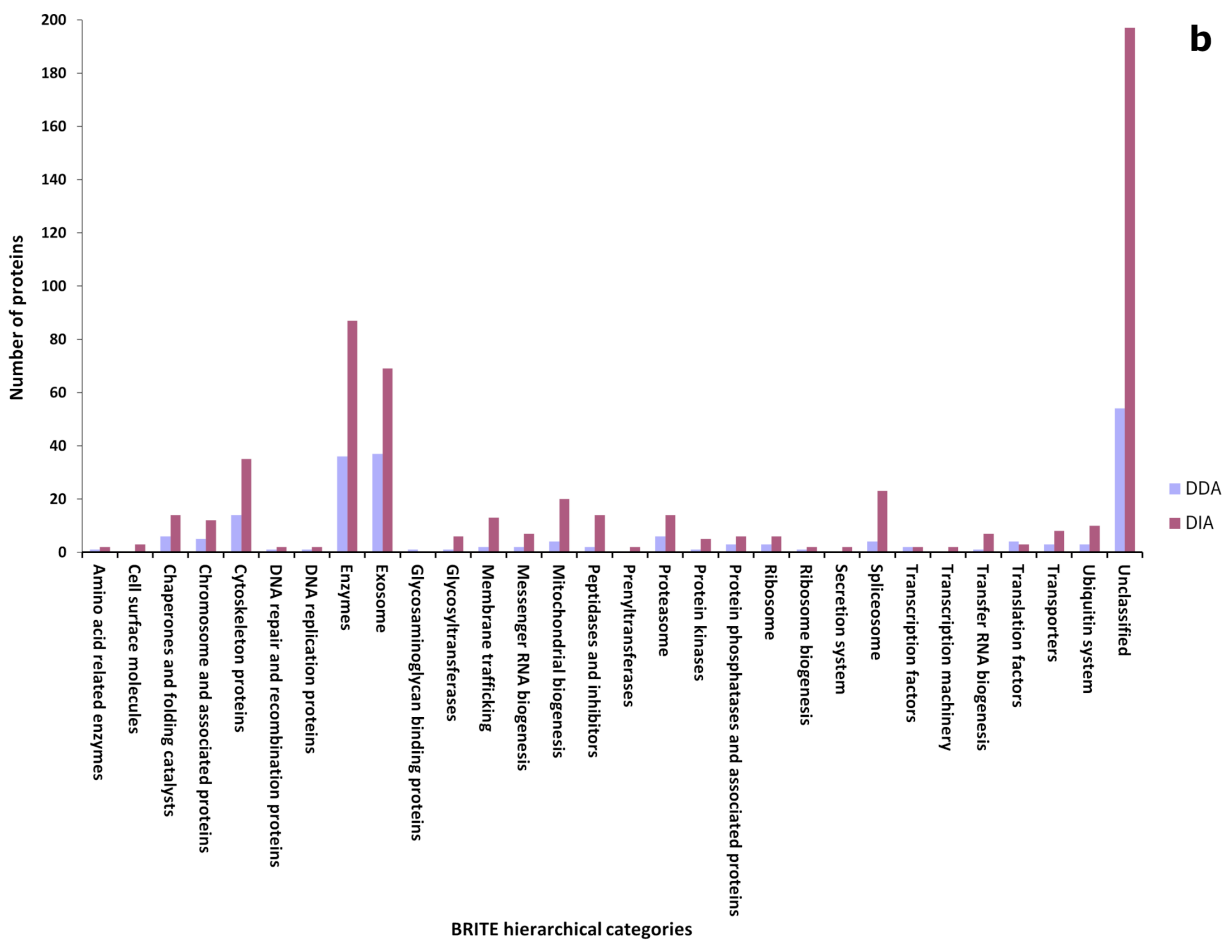

Supplement: Supplementary file 3 — Supplementary Material 3. [file 41598_2025_30920_MOESM3_ESM.pdf]
